# Therapy for *Mycobacterium kansasii* Infection: Beyond 2018

**DOI:** 10.3389/fmicb.2018.02271

**Published:** 2018-09-24

**Authors:** Michelle S. DeStefano, Carolyn M. Shoen, Michael H. Cynamon

**Affiliations:** ^1^Central New York Research Corporation, Syracuse, NY, United States; ^2^Veterans Affairs Medical Center, Syracuse, NY, United States

**Keywords:** *M. kansasii*, antimycobacterials, mouse models, non-tuberculous mycobacteria, pulmonary infection

## Abstract

The current standard of care therapy for pulmonary *Mycobacterium kansasii* infection is isoniazid (300 mg/day), rifampin (600 mg/day), and ethambutol (15 mg/kg/day) for 12 months after achieving sputum culture negativity. Rifampin is the key drug in this regimen. The contribution of isoniazid is unclear since its *in vitro* MICs against *M. kansasii* are near the peak achievable serum levels and more than 100-fold greater than the MICs for *Mycobacterium tuberculosis*. Ethambutol likely decreases the emergence of rifampin resistant organisms. There are several new drug classes (e.g., quinolones, macrolides, nitroimidazoles, diarylquinolines, and clofazimine) that exhibit antimycobacterial activities against *M. tuberculosis* but have not yet been adequately studied against *M. kansasii* infections. The evaluation of *in vitro* activities of these agents as well as their study in new regimens in comparison to the standard of care regimen in mouse infection models should be undertaken. This knowledge will inform development of human clinical trials of new regimens in comparison to the current standard of care regimen. It is likely that shorter and more effective therapy is achievable with currently available drugs.

## Introduction

*Mycobacterium kansasii* (*M. kansasii*) is a group I non-tuberculous mycobacterium (NTM) (Buhler and Pollack, 1953). These organisms are ubiquitous in the environment and are often found in aquatic settings (Amha et al., 2017). Seven genotypes, or subtypes have been identified, along with an intermediate (I/II) and atypical (IIb) subtype (Bakuła et al., 2018). Types I and II are the most common clinical subtypes found while types III-VII have generally only been recovered from environmental samples (Taillard et al., 2003).

*Mycobacterium kansasii* causes infection in both immunocompetent and immunosuppressed individuals. Additionally, it is the non-tuberculous mycobacterium most frequently found in immunocompetent patients. It is also the second most frequent mycobacterium found in HIV-infected patients and is only surpassed by *Mycobacterium avium* complex (Canueto-Quintero et al., 2003). The number of NTM infections is increasing and are geographically wide-spread. *M. kansasii* is the second most prevalent cause of NTM disease in the United States, China, South American countries, and some European countries such as Poland, Slovakia, and the United Kingdom (Hoefsloot et al., 2013).

*Mycobacterium kansasii* causes lung disease that clinically and radiologically resembles tuberculosis. The American Thoracic Society (ATS) and the Infectious Diseases Society of America (IDSA) recommend a combination of three anti-tuberculosis drugs, isoniazid (INH) at 300 mg/day, rifampin (RIF) at 600 mg/day, and ethambutol (EMB) at 15 mg/kg/day for treatment of *M. kansasii* pulmonary disease in HIV negative individuals. Treatment should be continued for 1 year after culture negativity (Griffith et al., 2007).

*Mycobacterium kansasii* infection is challenging to treat since the course of therapy requires multiple drugs and treatment periods of up to 2 years. Long periods of treatment often give rise to additional problems including patient non-adherence to the treatment plan, expense, and potential drug interactions and/or adverse events (Larsson et al., 2017). There are several antimycobacterial drugs that are effective against tuberculosis and other NTMs that might be better alternatives to the current three drug combination. The British Thoracic Society recommends the inclusion of a macrolide, such as azithromycin (AZI) at 250 mg/day or 500 mg of clarithromycin (CLA) twice a day as an alternative to INH (Haworth et al., 2017).

The focus of this review is to discuss *in vitro* susceptibility testing, *in vivo* evaluation in animal models, and clinical reports or studies utilizing these antimycobacterial agents. We will also discuss future efforts that should be undertaken to improve the clinical outcome of *M. kansasii* pulmonary infection.

## *In Vitro* Susceptibility Testing

*In vitro* susceptibility testing of *M. kansasii* to antimycobacterial agents has been accomplished using several different methodologies, including agar dilution, broth macrodilution or microdilution method, or BACTEC. Investigations to determine the minimal inhibitory concentration (MIC) of antimicrobials against *M. kansasii* have been performed on multiple clinical isolates. **Table 1** contains the MIC_50_ and MIC_90_ (defined as the lowest concentration at which 50 and 90% of the clinical isolates tested were inhibited, respectively) of drugs currently used to treat *M. kansasii* infection, as well as other drugs used to treat tuberculosis and some NTM infections. The list includes oxazolidinones, quinolones, macrolides, riminophenazines, diarylquinolines, nitroimidazoles, glycylcyclines, and rifamycins.

**Table 1:** MIC (μg/ml) of various compounds against *M. kansasii*.

Drug	MIC_50_	MIC_90_	Method	*N*	Reference
Linezolid	1	2	Broth microdilution	20	Shoen et al., 2018
Sutezolid	0.125	0.25	Broth microdilution	20	Shoen et al., 2018
Tedizolid	0.5	1.0	Broth microdilution	20	Shoen et al., 2018
Contezolid	1.0	1.0	Broth microdilution	20	Shoen et al., 2018
Isoniazid	0.5	16.0	Broth microdilution	169	Da Silva Telles et al., 2005
Ethambutol	16	16.0	Broth microdilution	169	Da Silva Telles et al., 2005
Clofazimine	0.5	2.0	Broth microdilution	169	Da Silva Telles et al., 2005
Bedaquiline	0.06	>16.0	Broth microdilution	84	Pang et al., 2017
Delamanid	0.025	0.1	Agar dilution	20	Doi and Disratthakit, 2006
Pretomanid	12.5	12.5	Agar dilution	20	Doi and Disratthakit, 2006
Clarithromycin	0.12	0.5	BACTEC	148	Alcaide et al., 2004
Azithromycin	8.0	8.0	BACTEC	19	Witzig and Fransblau, 1993
Moxifloxacin	0.06	0.06	BACTEC	148	Alcaide et al., 2004
Levofloxacin	0.12	0.12	BACTEC	148	Alcaide et al., 2004
Tigecycline	≤0.06	<0.12	Broth microdilution	11	Wallace et al., 2002
Rifampin	0.06	0.125	Broth microdilution	22	Cynamon and Sklaney, 2013
Rifabutin	0.004	0.015	Broth microdilution	22	Cynamon and Sklaney, 2013
Rifapentine	0.015	0.125	Broth microdilution	22	Cynamon and Sklaney, 2013

Throughout the literature there is a divergence in the MIC values for CLA and AZI. The reported MIC values of susceptible strains of *M. kansasii* for CLA are generally consistent with those listed in **Table 1** with the MIC_50_ 0.12 and MIC_90_ 0.5 μg/mL. Other MIC values reported are: 0.125–0.25 μg/mL (Griffith et al., 2003), MIC_50_ ≤ 0.06 MIC_90_ 0.125 μg/mL (Guna et al., 2005), MIC_50_ 0.25 and MIC_90_ 0.5 μg/mL (Witzig and Fransblau, 1993), and MIC_50_ 0.125, MIC_90_ 0.25 μg/mL (Brown et al., 1992). Although a more recent study (Li et al., 2016) suggests that CLA resistance is related to the Subtype I phenotype of *M. kansasii* and reports much higher MIC_90_ values (MIC_50_ 0.25, MIC_90_ 128 μg/mL). Subtype 1 is the most prevalent type of *M. kansasii* worldwide. In the same study the MIC values of AZI (MIC_50_ 16 MIC_90_ 32 μg/mL) are similar to those reported in **Table 1** (MIC_50_ 8 MIC_90_ 8 μg/mL). It is possible that these higher values are due to cross resistance with CLA as there are CLA resistant isolates identified in the same study. Other studies report MIC values of >8 μg/mL (Yew et al., 1994). Conversely, there are also reports of MIC values of AZI being similar to those seen with clarithromycin (Brown et al., 1992; Klemens and Cynamon, 1992).

The clinical MIC cutoff for EMB to define susceptibility/resistance against *M. kansasii* is 4 μg/ml, however, there is growing evidence that many *M. kansasii* isolates are resistant to EMB by this definition (**Table 1**). Patients diagnosed with this infection are not routinely evaluated for EMB susceptibility, therefore, resistance may be more prevalent than previously thought. It has been suggested that resistance to EMB (and INH) in *M. kansasii* occurs when a patient develops RIF resistance while on the standard regimen (Hjelm et al., 1992; Griffith et al., 2003). In a 2016 study, isolates belonging to *M. kansasii* Subtype I were found to exhibit greater resistance to EMB than the other subtypes (Li, 2016) although the differences were not statistically significant perhaps due to their small sample size. In a study looking at isolates from Poland, Germany, and Netherlands, 97.9% of the isolates were EMB resistant (all subtypes with the exception of Subtype V), however, published results from other countries indicates a resistance range for EMB of 0–94% as well as variable methods used to determine susceptibility (Bakula, 2018). This raises the question whether EMB and INH should be used as companion drugs to rifampin when newer, potentially more efficacious drugs are currently available.

A model of intracellular *M. kansasii* infection has been designed to mimic the pharmacodynamics and pharmacokinetics between infected macrophages and therapeutic agents (Srivastava et al., 2015; Srivastava and Gumbo, 2018). The hollow fiber model of *M. kansasii* has been adapted from similar models for *Mycobacterium tuberculosis* and *M. avium* complex (Deshpande et al., 2010; Srivastava and Gumbo, 2011). It has been reported that this system evaluates compound efficacy, rank orders kill rates compared to standard regimens, and assesses antibiotic tolerance by the use of efflux pump inhibitors. In the case where samples were available from moxifloxacin (MOX) treated patients, analysis of bronchial secretions, alveolar macrophages and lung epithelium lining fluid were used to simulate optimal doses and breakpoints for resistance with the aid of computer software (Srivastava et al., 2015). This preclinical disease model will provide valuable additional information to help select the appropriate agents for *in vivo* testing and perhaps subsequent evaluation in clinical studies.

The immune status of a patient determines the course of infection and the selection of an appropriate regimen. RIF is currently the cornerstone for the therapy of *M. kansasii* infection, but in patients co-infected with HIV, RIF presents a problem since it increases the hepatic metabolism of protease inhibitors, often used for the treatment of HIV infection (Brown-Elliott et al., 2012). Rifabutin (RBT) has less effect on the hepatic metabolism, therefore, it is often used as an alternative to RIF in HIV-infected patients. Rifapentine (RPT) is an alternative to RIF or RBT. All three rifamycins have demonstrated good *in vitro* activity against *M. kansasii* (Cynamon and Sklaney, 2013).

Linezolid (LZD), an oxazalidinone, is currently an option for multi-drug resistant tuberculosis infections and has been shown to have good *in vitro* activity against *M. kansasii* (**Table 1**), but there are significant toxicity issues with this drug. These toxicity issues make the use of this drug for *M. kansasii* therapy problematic. The newer oxazolidinones, tedizolid, and contezolid, have fewer side effects than LZD, and have good *in vitro* activity against *M. kansasii* (Shoen et al., 2018).

Four other drugs that are used to treat drug resistant tuberculosis have also shown good *in vitro* activity against *M. kansasii* (**Table 1**). They are clofazimine (CFZ) (riminophenazine), bedaquiline (diarylquinoline), delamanid (nitroimidazole), and pretomanid (nitroimidazopyran) although the MIC_50_ of pretomanid is significantly high and therefore may not be a viable candidate compound. Attempting to correlate *in vitro* efficacy with achievable serum levels is not always useful when selecting compounds for *in vivo* testing and treatment.

Several factors affecting serum levels and compound availability have to be considered. These include tissue/cell accumulation, interaction with other drugs, and length of treatment (single dosing versus multiple doses). CFZ illustrates the complexity of this issue. A single 200 mg dose in humans results in peak plasma levels of 0.41 μg/ml, which is lower than reported MIC values (**Table 1**), however, CFZ concentrates in macrophages and tissues long-term due to its highly lipophilic character (Cholo et al., 2012). INH treatment with CFZ increases the plasma concentration and reduces tissue concentration of CFZ (Haworth et al., 2017). Several studies with other antimycobacterial agents allude to a synergistic relationship with CFZ when evaluated in combination therapy against a variety of NTM species (McGuffin et al., 2017).

Since treatment for *M. kansasii* and other NTM infections consist of a multi-drug regimen, a comprehensive review of each compound’s known characteristics need to be considered to better predict potential efficacy. The established method for evaluation of *in vitro* combination therapy, “the checkerboard” has been used to define synergy or antagonism between two compounds. It is not clear whether results from this method correlate with those from *in vivo* modeling. New methods are being developed for prioritizing drug combinations, however, they have not yet been utilized for modeling regimens of *M. kansasii* therapy. Due to the lack of resources available for *M. kansasii* research it continues to be important to utilize available information on candidate compounds from *in vitro* combination studies focused on *M. tuberculosis* to advise our prioritization of regimens for *M. kansasii*.

## Animal Studies

Relatively little research has been done utilizing animal models to develop new therapies for NTM generally (Soni et al., 2016) and *M. kansasii* infection specifically. Research into the efficacy of compounds has largely been limited to drugs currently in use to treat tuberculosis or licensed drugs such as quinolones and macrolides. Due to the emerging importance of this infection, and the challenges of treatment for patients with resistant infections, more research into the development of an appropriate animal model to test new therapeutics is needed.

A review of the available literature indicates that several different strains of mice, levels of inocula, and routes of infection have been used, all with the ability to sustain a measurable bioburden. Presently there is no standard model and the strains of mice and routes of infection used are varied. Described below is a summary of the available literature on animal studies evaluating antimycobacterial compounds against *M. kansasii*.

BALB/c athymic (nude) mice infected with 10^7^ CFU *M. kansasii* intravenously resulted in disseminated infection (Graybill and Bocanegra, 2001). Untreated control mice succumbed to the infection after about 2 months. Therapy given daily for 21 days was started 1-week post-infection. Increasing doses of RPT (0.15, 0.3, and 0.6 mg/kg), AZI (10, 15, 30, 50, and 150 mg/kg) and EMB (10, 25, and 100 mg/kg) were evaluated in a survival study. RPT dosed at 0.6 mg/kg, AZI at 15 mg/kg, and EMB at 10 mg/kg or higher doses of each prolonged survival. There was no difference in survival between RPT and AZI. The investigators also evaluated bioburden reduction in the spleens and livers of mice treated with RPT and AZI alone and in combination. The results indicated that the combination was no more effective than RPT alone.

In another study, beige mice (C57BL/6J background) were infected intravenously with 10^7^ CFU of *M. kansasii* and treated 1-week post-infection, 5 days/week for 4 weeks. AZI 200 mg/kg, CLA 200 mg/kg, EMB 125 mg/kg, RIF 20 mg/kg, and CFZ 20 mg/kg were evaluated for activity compared to untreated controls. Viable cell counts were enumerated from the spleens and lungs. RIF reduced lung CFUs by about 1 log compared to the early controls and yielded the greatest reduction in organisms in the lungs. AZI and CLA had similar, but modest activity (Klemens and Cynamon, 1994).

In a study using beige mice infected intranasally with approximately 10^6^ CFU of *M. kansasii*, treatment with gatifloxacin (GAT) 100 mg/kg, CLA 200 mg/kg, LZD 100 mg/kg, RIF 20 mg/kg, GAT + CLA, CLA + LZD, GAT + LZD, and RIF + CLA was given for 4 weeks, 5 days/week. All of the treatment groups were significantly better than the early controls (evaluated at the initiation of therapy, 1-week post-infection). CLA was more effective than any other monotherapy. CLA combined with LZD, GAT, or RIF was better than any single drug therapy (Cynamon et al., 2003; **Table 2**).

**Table 2:** *In vivo* results of *M. kansasii* intranasal infection in mice.

Mouse strain	Inoculum	Drug regimen (n)	Log_10_ CFU ± S.D.
		Early controls (6)	7.47 ± 0.35
		Late controls (6)	8.70 ± 0.044
		Gatifloxacin 100 mg/kg (5)	6.50 ± 0.41
		Clarithromycin 200 mg/kg (5)	5.38 ± 0.64
Beige	1.4 × 10^6^ CFU	Gatifloxacin/Clarithromycin (6)	5.78 ± 0.35
		Rifampin 20 mg/kg (6)	6.30 ± 0.64
		Rifampin/Clarithromycin (5)	5.00 ± 0.13
		Linezolid 100 mg/kg (6)	7.03 ± 0.13
		Clarithromycin/Linezolid (6)	5.23 ± 1.02
		Gatifloxacin/Linezolid (6)	5.71 ± 1.27
Cynamon et al., 2003			

**Mouse strain**	**Inoculum**	**Drug regimen (n)**	**Median log counts^∗^**

		Early controls (8)	5.39 (5.00–5.72)
		Late controls (8)	6.45 (6.40–6.69)
		Azithromycin 100 mg/kg (7)	4.95 (4.76–5.04)
C57BL/6J	8 × 10^4^ CFU	Rifalazil 10 mg/kg (8)	2.86 (2.21–3.12)
		Rifalazil/Azithromycin (5)	2.11 (1.48–2.70)
		Rifampin 10 mg/kg (8)	5.79 (5.53–6.23)
		Rifampin/Azithromycin (7)	4.40 (4.15–4.89)
Cynamon and Buswell, 2002			

**Mouse strain**	**Inoculum**	**Drug regimen (n)**	**Median log counts^∗^**

		Early controls (5)	6.94 (6.23–7.08)
		Late controls (7)	7.73 (7.56–8.01)
		Rifalazil 1 mg/kg (6)	6.29 (5.85–6.45)
C57BL/6J	4.4 × 10^5^ CFU	Rifalazil 5 mg/kg (8)	4.76 (4.64–4.92)
		Rifalazil 10 mg/kg (8)	2.18 (1.95–2.45)
		Rifampin 20 mg/kg (7)	5.45 (5.13–5.72)
		Gatifloxacin 100 mg/kg (7)	4.48 (4.22–4.68)
Cynamon and Monica, 2003			

*^∗^95% confidence interval.*

More recent studies with immunocompetent C57BL/6J mice demonstrate that *M. kansasii* can also survive and replicate in these mice. Two studies were carried out where mice were infected with approximately 5 × 10^5^ CFU/mouse via the intranasal route. In the first experiment, RIF 10 mg/kg, rifalazil (RZL) 10 mg/kg, AZI 100 mg/kg and combinations of RIF + AZI and RZL + AZI were evaluated. All treatment groups were significantly better than the early controls. The combination of RZL + AZI was the most effective treatment group (Cynamon and Buswell, 2002; **Table 2**). In the second experiment, 4 weeks (5 days/week) of GAT 100 mg/kg and RIF 20 mg/kg were compared to increasing doses of RZL (1, 5, and 10 mg/kg). The individual drugs were also compared to a combination of GAT + RZL10 mg/kg. All single agents and the combination regimen had significant activity compared to the early controls (evaluated 1-week post-infection). There was a dose response with RZL and the combination of RZL + GAT reduced lung CFUs to an undetectable level after 4 weeks of therapy (Cynamon and Monica, 2003; **Table 2**).

Although the published literature is limited regarding the use of *M. kansasii* infection models as a screening tool to evaluate recently licensed or new experimental compounds, the above studies suggest that these models should be used to determine if more effective therapy is achievable and whether their use may lead to a shorter duration of therapy compared to the current standard regimen.

## Clinical Research

Rifampin, INH, and EMB (ATS/IDSA) or RIF, CLA (or AZI), EMB (British Thoracic Society) are suggested regimens for treatment of *M. kansasii* infection in humans taking between 12–18 months depending on the sputum culture time of conversion. The standard treatment regimen is effective in most instances; however, therapy is lengthy and it does not provide a cure for all patients (Santin et al., 2009). Treatment failure is almost always associated with resistance to RIF and/or CLA (Brown-Elliott et al., 2012). The Clinical and Laboratory Standards Institute (CLSI) currently recommends testing all initial patient isolates of *M. kansasii* for RIF and CLA *in vitro* susceptibility. Broth dilution is the suggested methodology utilizing cation-adjusted Mueller Hinton broth and 5% OADC (oleic acid, albumin, dextrose, catalase) enrichment as the media (Griffith et al., 2007). When isolates are RIF resistant (MIC > 1 μg/ml), the suggestion is to test secondary agents such as AMK, EMB, LZD, MOX, RBT, and trimethoprim-sulfamethoxazole for *in vitro* activity (Brown-Elliott et al., 2012).

The current method for testing and interpretation of the MIC for INH in the clinical laboratory utilizes the critical concentrations (0.2 μg/ml, 1 μg/ml) for *M. tuberculosis* susceptibility testing. This results in a false interpretation of INH resistance because the MIC values for *M. kansasii* usually range from 0.5 μg/ml to 5 μg/ml (Brown-Elliott et al., 2012). Heifets’ work illustrated this issue stating that of more than 100 *M. kansasii* isolates evaluated by agar dilution methodology almost all were completely resistant to 0.2 μg/ml and susceptible, or partially resistant to 1 μg/ml of INH (Heifets, 1991). MIC testing of INH is not recommended.

Although infection can occur in other organs or become disseminated, the focus of our literature review of clinical outcomes is pulmonary infection caused by *M. kansasii.* A systematic review of microbiological and clinical treatment outcomes for *M. kansasii* pulmonary infections highlighted the lack of randomized controlled clinical trials for this infection (Diel et al., 2017; Haworth et al., 2017). A small cohort of prospective and retrospective observational studies exist and a few representative studies are discussed below.

A prospective study by the British Thoracic Society evaluated 173 patients with pulmonary *M. kansasii* infection. Prior to species identification, 149 of these patients were treated with regimens of INH + RIF + EMB, INH + RIF + EMB + PZA, or INH + RIF + PZA + streptomycin. Once *M. kansasii* was identified patients were given RIF + EMB for an additional 9 months (Jenkins et al., 1994). The remaining 24 patients received 9 months of RIF + EMB. Their results suggested that the RIF + EMB was acceptable treatment for non-immune compromised patients. Only 11% of their patients had positive sputum cultures after 3 months of therapy. Fifteen patients (9.7%) of the 154 patients who entered the post-chemotherapy follow up period relapsed between 6 and 50 (median 23) months.

In a retrospective study of 111 patients diagnosed with *M. kansasii* lung disease and treated with RIF + EMB + INH supplemented with streptomycin during the initial 2–3 months, 75 patients completed 12 months of therapy (Santin et al., 2009). After a 41.5-month median follow-up, five (6.6%) patients relapsed. The authors concluded that a 12-month course is adequate for most, but not all patients.

A prospective trial by Griffith et al. (2003) evaluated a thrice-weekly regimen of CLA 1000 mg (two female patients who weighed <50 kg received 500 mg), EMB 25 mg/kg, and RIF 600 mg in 18 patients with *M. kansasii* lung disease. This regimen yielded a durable cure with no relapse after a follow-up of 46 ± 8 months (mean time ± standard deviation) for the 14 patients that completed therapy. Four patients were lost to follow-up. The mean time to sputum conversion was 1 ± 0.9 months and the mean duration of therapy was 13.4 ± 0.9 months (Griffith et al., 2003).

## Future Efforts

The efficacy of INH when added to RIF + EMB should be measured in an appropriate mouse model to evaluate the contribution of INH to the standard regimen. New drug regimens should be tested in a mouse model of *M. kansasii* infection and compared to RIF + EMB ± INH to determine comparative efficacy. Similar to *in vitro* evaluation, multiple clinical isolates of *M. kansasii* should be studied in a mouse model to ensure that isolate differences do not affect efficacy in the mouse model. It is preferable to deliver the inoculum by the aerosol or intranasal route since this method most closely parallels the human pulmonary route of infection.

Initially, 4 weeks of treatment should be used to determine which regimens to evaluate in more detail (i.e., longer duration of therapy followed by a non-treatment observation period). It is yet to be determined whether one particular mouse strain is preferable to another. It would be of interest to evaluate C3HeB/FeJ mice as a potential model system for *M. kansasii* infection since they develop lesions similar to humans when infected with *M. tuberculosis* (Driver et al., 2012; Harper et al., 2012).

Several agents included in **Table 1** (bedaquiline, delamanid, MOX, tedizolid, contezolid, CFZ, and CLA) should be studied in a mouse model of *M. kansasii* infection to determine whether they can improve outcomes with regard to efficacy and duration of therapy. CFZ added to INH + RIF + PZA in mice infected with tuberculosis was shown to decrease the time needed to achieve a durable cure (Tyagi et al., 2015; Ammerman et al., 2018). It should be determined whether CFZ has a similar effect with RIF + EMB or in the case of possible RIF resistance, a combination of CFZ with 2 other drugs in *M. kansasii* infections.

Several tuberculosis clinical trials with RIF suggest that doses of 20 and 35 mg/kg are both well tolerated and more efficacious than the standard 10 mg/kg dose (600 mg daily) (Tiberi et al., 2018). Higher doses of RIF should be evaluated to assess whether this treatment is also beneficial in reducing the lung bioburden of *M. kansasii* infected mice.

In order to accomplish randomized controlled clinical trials evaluating new regimens for therapy of pulmonary *M. kansasii* infection it is necessary to develop a clinical trials consortium, similar to the Mycoses Study Group^1^. This would enable multiple clinical trial sites to recruit and enter patients into clinical trials. In 2009 the European based MTB clinical trial consortium TBnet, formed the Non-tuberculous Mycobacteria Network European Trials Group (NTM-NET). NTM-NET is an international consortium of clinical NTM researchers that is primarily focused in Europe^2^ and is able to provide limited funding for NTM research. Perhaps now is an appropriate time for a Non-tuberculous Mycobacteria Study Group funded by NIAID to advance clinical trials for NTM infections by providing opportunities and resources for this important work.

## Author Contributions

MD, CS, and MC have equally contributed to the research of the content, writing, and editing of this article. All authors have approved the final version of the article.

## Conflict of Interest Statement

The authors declare that the research was conducted in the absence of any commercial or financial relationships that could be construed as a potential conflict of interest.

## Footnotes

1 http://www.msgerc.org

2 http://www.ntm-net.org
